# Regenerative responses to liver injury

**DOI:** 10.1186/2047-783X-19-S1-S1

**Published:** 2014-06-19

**Authors:** George K Michalopoulos

**Affiliations:** 1 Department of Pathology, University of Pittsburgh, School of Medicine, Pittsburgh, Pennsylvania, 15261, USA

## Introduction

The recent three decades emphasized research on liver regeneration, which focused on signals leading hepatocytes into proliferation and liver into regeneration. Mitogenic growth factors (ligands of EGFR and HGF) were identified as direct mitogens for both hepatocyte cultures as well as whole animals *in vivo*. Multiple other signals (including norepinephrine, IL6, TNF, bile acids, etc.) also initiate signaling pathways that streamline and optimize signaling pathways inside hepatocytes so that hepatocytes enter into cell cycle and proceed with DNA synthesis. Hepatocytes in the cell cycle generate paracrine signals to other hepatic cell types, producing GM-CSF (affecting Kupffer cells), VEGF-A and Angiopoietins 1 and 2 (affecting endothelial cells that themselves also produce HGF), TGFα and FGF1 (affecting a variety of cell types including hepatocytes themselves). Non-parenchymal cells in the liver also produce an array of mitogenic signaling molecules, including HGF (from stellate cells and endothelial cells), IL6 and TNF (from Kupffer cells). TGFβ1, considered as being produced primarily by stellate cells, does not appear to exert mito-inhibitory effects on regenerating hepatocytes, primarily due to down-regulation of TGFβ receptors during regeneration. Functions served by TGFβ during regeneration are more probably related to control of synthesis of hepatic biomatrix, which is substantially downgraded and remodeled during the early stages of regeneration and is resynthesized towards the end of the process [1]. Much less attention has been paid to the pathways leading to cessation of regeneration. It is remarkable that at the end of regeneration, liver returns to 100% of the original mass, suggesting the presence of a “hepatostat” correlating liver size to body size and function. Crucial to understanding this system is the understanding of the signaling pathways leading to proper termination of liver regeneration, as it is this phase of the process that guarantees return to the original size.

Many studies with a variety of epithelial cell types have shown elimination of growth suppressing signals in neoplastic development. Cancer tissues proceed with continual proliferation, due to elimination of internal checkpoints and cell cycle control systems.

In this study, we concentrated on several systems apparently associated with termination of liver regeneration and demonstrated that such systems are either non-functional or eliminated in hepatocellular carcinomas (HCC). Conversely, by applying detailed genomic analysis, we also found specific genes deleted in the majority or in many hepatocellular carcinomas whose function, not apparent at first, appears to relate with growth suppressor signals associated with termination or negative control of liver regeneration. Such signals related to the present study are as follows:

## Glypican 3 (GPC3)

This protein is massively upregulated in HCC, to the point that it is used as a diagnostic tool. While it should be intuitively considered as enhancing cell growth, loss of function of GPC3 is associated with organ overgrowth (human Simpson Golabi Behmel syndrome). Transgenic overexpression of GPC3 in hepatocytes leads to deficient liver regeneration and smaller livers, with lower nuclear levels of protein Yap [2]. GPC3 is a GPI-linked protein with no apparent intracellular signaling domain. It binds to a tetraspanin known as CD81 [3]. The latter is the first protein to which HCV attaches in order to enter into hepatocytes, HCV entry also being facilitated by other proteins including members of the Claudin and Occludin family, interacting with the complex of the CD81 and the E2 protein of HCV. We identified GPC3 and the protein known as Hhex (associated with liver development) as proteins binding to CD81 by a yeast-2 hybrid assay. Following detailed studies of protein interactions at different stages of liver regeneration, we now understand that in normal resting liver GPC3 binds to members of the Hedgehog family and prevents their interaction with the Patched-1 receptor. GPC3 also binds to CD81. Following partial hepatectomy, GPC3 ceases binding to Hedgehog and to CD81. Hhex translocates from the nucleus and now binds to CD81, thus being removed from exercising growth inhibitory effects [4]. The combination of these events releases inhibitory signals of Hhex and allows stimulatory signals from Hedgehog. The findings put into the context of liver regeneration a finding first identified from gene expression studies of liver cancer [5].

## Lymphocyte specific protein 1 (LSP1)

We identified this protein as having the highest number of gene deletions in a high detail copy number variation (CNV) analysis of human HCC [6]. There were 46/98 cases with deletions of LSP1 from the carboxyterminal region. Five cases (5/98) had amplifications in the carboxyterminal region, suggesting the creation of a decoy protein preventing the binding of the full protein to its target. LSP1 binds to a protein KSR which in turn holds together Raf, MEK and ERK, whose combined signal cascade leads to very important mitogenic signaling for most cell types [7]. Mice with genetic deletion of LSP1 have accelerated wound healing, suggesting suppressor effects of LSP1 on processes such as cell growth and migration. We have demonstrated that LSP1 expression increases at the end of liver regeneration. Inhibition of expression (RNA silencing) of LSP1from the JM1 rat hepatoma cell line led to dramatic increase in rate of cell migration, expression of cyclin D1 and cell proliferation. Conversely, over-expression of LSP1 in JM2 hepatoma cells, which do not express LSP1, led poor cell growth. We are exploring the functions of LSP1 in the context of regulation of hepatocyte growth and termination of liver regeneration. This is another study, which put into the context of liver regeneration a finding first identified from genomic studies of liver cancer [6].

## Rsu-1 and the Integrin Linked Kinase (ILK) associated IPP complex

ILK binds to β1 integrin chain and transmits a variety of growth suppressing signals in hepatocytes and many other epithelial cells types. We and others have shown that extracellular matrix (being remodeled and degraded at the early stages of liver regeneration) has growth-inhibitory effects on hepatocytes. Genetic elimination of ILK specifically from hepatocytes leads to enhanced spontaneous hepatocyte proliferation, enhanced deposition of extracellular matrix and larger livers (approximating 2-fold increase over normal). ILK genetic elimination from hepatocytes is also associated with abnormal enhanced liver weight (beyond the 100% of the original) at the end of regeneration after partial hepatectomy [8]. ILK binds to proteins α-Parvin and PINCH (IPP complex). The latter binds to another protein known as “Ras suppressor 1” (Rsu-1). The above studies were conducted in mice. In the same CNV study of human HCC mentioned above [6], we found that Rsu-1, identified as an IPP complex associated protein from the mouse studies, was also associated with deletions in 7/98 cases of human HCC. In subsequent studies we have now shown that Rsu-1 is not a direct suppressor of Ras function. It inhibits Rho kinases downstream of Ras, and its elimination should be a growth-promoting effect for HCC. This is a study in which findings associated with liver regeneration in rodents are now directly associated with genomic findings in human liver cancer.

Regenerative responses to liver injury have become a good source of identifying extracellular and intracellular signaling pathways that have relevance to growth regulation of most other normal and neoplastic cells.
